# The mutual interactions among Helicobacter pylori, chronic gastritis, and the gut microbiota: a population-based study in Jinjiang, Fujian

**DOI:** 10.3389/fmicb.2024.1365043

**Published:** 2024-02-14

**Authors:** Hanjing Li, Yingying Hu, Yanyu Huang, Shanshan Ding, Long Zhu, Xinghui Li, Meng Lan, Weirong Huang, Xuejuan Lin

**Affiliations:** ^1^College of Traditional Chinese Medicine, Fujian University of Traditional Chinese Medicine, Fuzhou, China; ^2^Research Base of Traditional Chinese Medicine Syndrome, Fujian University of Traditional Chinese Medicine, Fuzhou, China; ^3^Key Laboratory of Traditional Chinese Medicine Health Status Identification, Fuzhou, China; ^4^Jinjiang Hospital of Traditional Chinese Medicine Affiliated to Fujian University of Traditional Chinese Medicine, Jinjiang, China

**Keywords:** chronic atrophic gastritis, gut microbiota, Helicobacter pylori, gastritis, gastric microenvironment

## Abstract

**Objectives:**

Helicobacter pylori (*H. pylori*) is a type of bacteria that infects the stomach lining, and it is a major cause of chronic gastritis (CG). *H. pylori* infection can influence the composition of the gastric microbiota. Additionally, alterations in the gut microbiome have been associated with various health conditions, including gastrointestinal disorders. The dysbiosis in gut microbiota of human is associated with the decreased secretion of gastric acid. Chronic atrophic gastritis (CAG) and *H. pylori* infection are also causes of reduced gastric acid secretion. However, the specific details of how *H. pylori* infection and CG, especially for CAG, influence the gut microbiome can vary and are still an area of ongoing investigation. The incidence of CAG and infection rate of *H. pylori* has obvious regional characteristics, and Fujian Province in China is a high incidence area of CAG as well as *H. pylori* infection. We aimed to characterize the microbial changes and find potential diagnostic markers associated with infection of *H. pylori* as well as CG of subjects in Jinjiang City, Fujian Province, China.

**Participants:**

Enrollment involved sequencing the 16S rRNA gene in fecal samples from 176 cases, adhering to stringent inclusion and exclusion criteria. For our study, we included healthy volunteers (Normal), individuals with chronic non-atrophic gastritis (CNAG), and those with CAG from Fujian, China. The aim was to assess gut microbiome dysbiosis based on various histopathological features. QIIME and LEfSe analyses were performed. There were 176 cases, comprising 126 individuals who tested negative for *H. pylori* and 50 who tested positive defined by C14 urea breath tests and histopathological findings in biopsies obtained through endoscopy. CAG was also staged by applying OLGIM system.

**Results:**

When merging the outcomes from 16S rRNA gene sequencing results, there were no notable variations in alpha diversity among the following groups: Normal, CNAG, and CAG; OLGIM I and OLGIM II; and *H. pylori* positive [Hp (+)] and *H. pylori* negative [Hp (–)] groups. Beta diversity among different groups show significant separation through the NMDS diagrams. LEfSe analyses confirmed 2, 3, and 6 bacterial species were in abundance in the Normal, CNAG, and CAG groups; 26 and 2 species in the OLGIM I and OLGIM II group; 22 significant phylotypes were identified in Hp (+) and Hp (–) group, 21 and 1, respectively; 9 bacterial species exhibited significant differences between individuals with CG who were Hp (+) and those who were Hp (–).

**Conclusion:**

The study uncovered notable distinctions in the characteristics of gut microbiota among the following groups: Normal, CNAG, and CAG; OLGIM I and OLGIM II; and Hp (+) and Hp (–) groups. Through the analysis of *H. pylori* infection in CNAG and CAG groups, we found the gut microbiota characteristics of different group show significant difference because of *H. pylori* infection. Several bacterial genera could potentially serve as diagnostic markers for *H. pylori* infection and the progression of CG.

## Introduction

The gut microbiota comprises an intricate ecosystem teeming with tens of trillions of microorganisms (Lozupone et al., 2012; Sender et al., 2016). Throughout an individual's entire lifespan, the human gut microbiota participates in numerous interactions that impact the host's overall wellbeing (Milani et al., 2017). Disruption of gut microbiota homeostasis could correlate with alimentary tract injury (Chen et al., 2020), gastritis (Miao et al., 2022), and even gastric cancer (GC; Barra et al., 2021). The colonization of Helicobacter pylori (*H. pylori*) has a substantial impact on the gastric microenvironment, subsequently leading to alterations in the gastric microbiota (Chen et al., 2021). *H. pylori* infection is widely recognized for its links to gastroduodenal ulcer disease, chronic atrophic gastritis (CAG), GC, gastric intestinal metaplasia (GIM), and various other gastric and extragastric conditions (Watari et al., 2014; Fischbach and Malfertheiner, 2018). The interplay between *H. pylori* and the commensal flora in the gastrointestinal tract might contribute to *H. pylori*-related cancer risk and extragastric symptoms (Chen et al., 2021).

CAG is a type of gastritis, which is an inflammation of the stomach lining (Li et al., 2018). It is called “chronic” because it develops gradually over an extended period and “atrophic” because it is marked by the gradual depletion of gastric glandular cells, which are gradually replaced by fibrous tissue. One of the main causes of CAG is the presence of *H. pylori* infection (Holleczek et al., 2020; Yang et al., 2021; Zheng et al., 2023), which is a type of bacteria that can infect the stomach lining and cause chronic inflammation. Over time, as the inflammation persists, the gastric glands that produce hydrochloric acid and digestive enzymes start to deteriorate, leading to a reduction in the production of these essential substances (Neumann et al., 2013; Lahner et al., 2018). As a result, the stomach's ability to digest food and absorb certain nutrients, such as vitamin B12 and iron, is impaired. Subsequently, a range of gastrointestinal symptoms manifested, such as gas, upper abdominal discomfort, bloating, and changes in bowel movements. CAG can give rise to a range of health complications. Prolonged, persistent atrophic gastritis, particularly when linked with a chronic *H. pylori* infection, can elevate the likelihood of developing gastric (stomach) cancer (Karaman et al., 2008; Huang et al., 2023). But not everyone with CAG will experience noticeable symptoms, and in some cases, it may remain asymptomatic for an extended period (Liou et al., 2020). It is essential to manage CAG appropriately to prevent or minimize these potential morbidities.

It's encouraged for individuals with CAG to undergo regular screenings and monitoring for any signs of cancerous changes in the stomach lining. Diagnosing CAG usually involves a thorough assessment of the patient's medical history, blood tests, a physical examination to detect anemia or nutritional deficiencies, and endoscopy to visualize the stomach lining and collect biopsy samples for laboratory analysis (Shah et al., 2021). Diagnosing atrophic gastritis histopathologically continues to be challenging, characterized by limited agreement among different observers (Isajevs et al., 2014), and histopathological diagnosis is invasive. These all prompted us to find more convenient and accurate methods to find CAG patients. China exhibits a clear geographical clustering in the distribution of CAG and GC, with Fujian province standing out as a high-incidence area for this condition in the country (Li et al., 1996; Yang, 2006). The *H. pylori* infection is an important risk factor for CAG, affecting approximately half of the world's population, but the prevalence varies in different geographic regions (such as coastal, high altitude, and jungle; 1992; Yin et al., 2022; Malfertheiner et al., 2023). *H. pylori* infection is highly prevalent in coastal areas of China, such as Hainan and Shenzhen, with a high incidence rate among asymptomatic individuals (Li et al., 2022; Chen et al., 2023). Jinjiang City, located in the southeast of Fujian Province, China, is a typical coastal city, which has a certain representation of the population characteristics in coastal areas. This investigation employed 16S rRNA gene sequencing to investigate alterations in the ecological and composition aspects of gut microbiota for subjects from Jinjiang City, Fujian Province, aiming to understand their contributions to the transition from a healthy state to chronic non-atrophic gastritis (CNAG), followed by CAG, and their connection to *H. pylori* infection. To further identify potential diagnostic markers for *H. pylori* infection and the progression of chronic gastritis (CG).

## Materials and methods

### Study subjects

**Figure 1:**
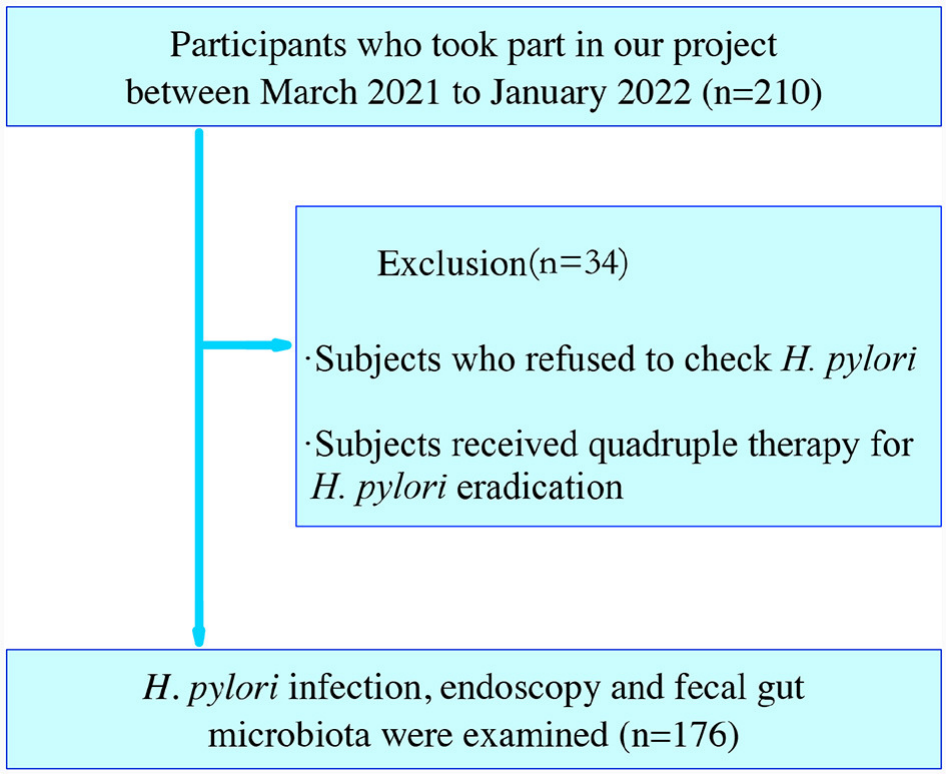
Flowchart of time and population included in the study.

This research enrolled 210 adult participants who had undergone a health assessment at the Spleen and Stomach Department and Health Management Center of Jinjiang Hospital of Traditional Chinese Medicine affiliated to Fujian University of Chinese Medicine, Jinjiang City, Fujian, China, between March 2021 to January 2022. The diagnosis of CAG (including OLGIM I and OLGIM II) and CNAG was evaluated by physicians based on a combination of endoscopic information and histopathological results. *H. pylori* positive [Hp (+)] and *H. pylori* negative [Hp (–)] was defined by C14 urea breath tests or biopsies. Healthy controls (Normal) had no self-report and physician diagnosis of past or current gastritis. Exclusion criteria encompassed individuals with a pacemaker, defibrillator, or other metallic implants; gastrointestinal tract obstruction; poor general condition; dysphagia or delayed gastric emptying; pregnancy. Out of these, we excluded 34 participants who refused to check *H. pylori* or received quadruple therapy for *H. pylori* eradication, including proton pump inhibitors (PPI) or antibiotic treatment. Finally, 176 subjects were analyzed (Figure 1).

### Ethical approval and participant consent

This study adhered to the Declaration of Helsinki and received approval from the ethics committee of Jinjiang Hospital of Traditional Chinese Medicine, affiliated with Fujian University of Traditional Chinese Medicine Medical Ethics Committee (2019-016). We obtained written informed consent from all patients who participated.

### Diagnosis of *H. pylori* infection

C14 urea breath tests or biopsies obtained through endoscopy were used to diagnose *H. pylori* infection. C14 breath tests were subjected after more than 6 h-fasting. An *H. pylori* standard value exceeding 100 (dpm/mmol) indicated a positive outcome, while a negative outcome corresponded to a range of 0–100 (dpm/mmol). In most instances, the antrum served as the primary site for biopsy to detect *H. pylori* infection. Nevertheless, in order to mitigate the potential for false negative outcomes in individuals with intestinal metaplasia (IM) or antral atrophy, a biopsy from the corpus at the greater curve was utilized. Additionally, *H. pylori* infection was diagnosed in real-time during the endoscopic examination.

### Evaluation of atrophic gastritis

Characteristic endoscopic signs of atrophic gastritis consist of a pale appearance of the gastric mucosa, enhanced visibility of vasculature due to mucosal thinning, and the absence of gastric folds. In cases where light blue crests and white opaque fields were present, a concurrent diagnosis of intestinal metaplasia might be established. According to the Sydney System (Dixon et al., 1996), two endoscopists with more than 5 years' experience in gastroscopy performed five biopsies: two antral and two corpus, a fifth or more biopsy specimens from the incisura as a supplement. Biopsy samples from the three regions were individually distinguishable upon submission to the laboratory. Both a general and a specialist gastrointestinal pathologist evaluated and graded the biopsy specimens using Sydney classification, and the gastritis stage was evaluated based on the OLGIM system. Combined endoscopic information with histopathology, subjects were divided into CNAG, and CAG. In the OLGIM staging system, the evaluation of IM followed a similar scoring approach to that of OLGA. However, instead of the atrophy score, it was replaced with an IM score (based on the presence of IM; Capelle et al., 2010). We then categorized the subjects into OLGIM I and OLGIM II groups.

### Extraction of DNA from fecal samples

Each participant collected 2–3 g of fecal samples in commercial containers (Sorfa Life Science Research Co., Ltd., Zhejiang, China) and suspended in GUHE Flora Storage buffer (Zhejiang Hangzhou Equipment Preparation 20190682, GUHE Laboratories, Hangzhou, China). Preserved the collected samples at −80°C before the procedure of DNA extraction, and all samples were maintained under identical storage conditions.

The GHFDE100 DNA isolation kit (Zhejiang Hangzhou Equipment Preparation 20190952) was used to obtain genomic DNA from bacteria. The extraction procedure adhered to the guidelines of manufacturer. Agarose gel electrophoresis and NanoDrop ND-1000 spectrophotometer (Thermo Fisher Scientific, Waltham, MA, USA) were used to ensure the quality and quantity of the obtained DNA.

### Analysis of next-generation sequences and taxonomy based on 16S rDNA

The V4 region of bacterial 16S rRNA genes were amplified via PCR, employing the forward primer 515F (5′-GTGCCAGCMGCCGCGGTAA-3′) and the reverse primer 806R (5′-GGACTACHVGGGTWTCTAAT-3′). The PCR amplicons were subsequently purified with Agencourt AMPure XP Beads from Beckman Coulter, Indianapolis, IN, and their quantification was determined using the PicoGreen dsDNA Assay Kit from Invitrogen, Carlsbad, CA, USA. After quantifying each amplicon individually, they were mixed in equal proportions and then underwent pair-end 2 × 150 bp sequencing on the Illumina NovaSeq6000 platform at GUHE Info Technology Co., Ltd in Hangzhou, China. We employed the following criteria to filter out low-quality sequences (Gill et al., 2006): sequences shorter than 150 base pairs, sequences with mononucleotide repeats longer than 8 base pairs, sequences with an average Phred score below 20, and sequences containing ambiguous bases were all excluded. The paired-end reads were merged using Vsearch V2.4.4 (-fastq_mergepairs–fastq_minovlen 5), and then operational taxonomic units (OTUs) were determined using Vsearch v2.15.0. The taxonomic classification of the OTU was conducted (Quast et al., 2013) and an OTU table was created to document the abundance of each OTU in every sample, along with their taxonomic assignments. Eliminating OTUs representing <0.01‰ of the total sequences. To ensure uniform sequencing depth across samples, we generated an OTU table by computing the averages of 100 evenly resampled OTU subsets. Each subset was resampled to meet 90% of the minimum sequencing depth.

QIIME2 was used to analyze the microbiome sequence data. OTUs were constructed using a >97% similarity threshold to the 16S rRNA representative sequence provided in the Green Genes Database v13.8.0. Alpha diversity, beta diversity and LEfSe analyses were performed.

### Statistical analysis

We used SPSS 26.0 to analyze clinical data. Categorical variables were presented as percentages and frequencies. Continuous variables were presented using either the mean and standard deviation or the median and interquartile range. We utilized the chi-square test for categorical variables and Student's *t*-test for continuous variables. One-way ANOVA was applied for two or more groups' comparison. Between-group effects were tested pairwise (two-tailed *t*-tests, alpha = 0.05). *P* < 0.05 was considered as a significance level.

## Results

### Characteristics of the subjects

One hundred and seventy-six subjects included 23 healthy volunteers (normal), 48 CNAG patients, and 105 CAG patients. Of all the patients with CAG, 52 subjects were classified as OLGIM I and 53 OLGIM II. A combined 126 subjects were Hp (–), while 50 Hp (+). By comparing general features of the 176 participates, we found the age of subjects increased from group normal, CNAG to CAG, and the disparity was statistically noteworthy (*P* < 0.01; Table 1). Comparing the occupations of participants in group OLGIM I with OLGIM II and group Hp (+) with Hp (–), it was observed that there were more farmers in group OLGIM II and Hp (+) (*P* < 0.05; Tables 2, 3). No significant distinctions were observed among the subjects of lifestyle including smoking and drinking (*P* > 0.05; Tables 1–3).

**Table 1:** General characteristics for the 176 participates who enrolled in this study.

**Items**	**Normal (*n* = 23)**	**CNAG (*n* = 48)**	**CAG (*n* = 105)**	***P*-value**
Sex				0.648
Female	12 (52%)	22 (46%)	44 (42%)	
Male	11 (48%)	26 (54%)	61 (58%)	
Age	40.044 ± 8.636	43.458 ± 9.704	49.971 ± 11.182	0.000013
Farmers	4 (17%)	22 (46%)	41 (39%)	0.066
Smoking	3 (13%)	13 (27%)	24 (23%)	0.417
Drinking	4 (17%)	10 (21%)	22 (21%)	0.926

**Table 2:** General characteristics for the 105 CAG patients who classified as OLGIM I and OLGIM II.

**Items**	**OLGIM I (*n* = 52)**	**OLGIM II (*n* = 53)**	***P*-value**
Sex			0.632
Female	23 (44%)	21 (40%)	
Male	29 (56%)	32 (60%)	
Age	48.40 ± 11.517	51.51 ± 10.729	0.369
Farmers	14 (27%)	27 (51%)	0.012
Smoking	12 (23%)	12 (23%)	0.958
Drinking	11 (21%)	11 (21%)	0.96

**Table 3:** General characteristics for the 176 participates who defined infection of *H. pylori*.

**Items**	***H. pylori* negative (*n* = 126)**	***H. pylori* positive (*n* = 50)**	***P*-value**
Sex			0.536
Female	54 (43%)	24 (48%)	
Male	72 (57%)	26 (52%)	
Age	47.66 ± 11.239	44.98 ± 10.752	0.622
Farmers	39 (31%)	28 (56%)	0.002
Smoking	26 (21%)	11 (22%)	0.841
Drinking	22 (17%)	10 (20%)	0.694

### Attributes of the 16S rRNA gene sequencing findings

**Figure 2:**
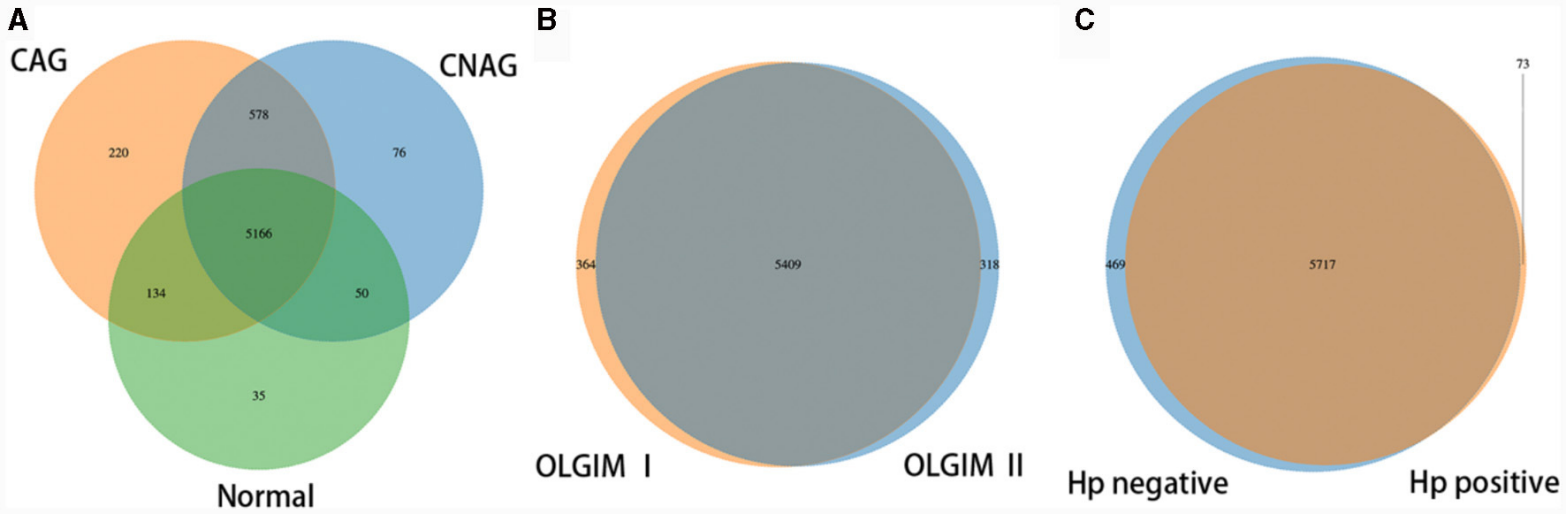
Venn map. **(A)** The Venn map of the OTUs distribution of group Normal, CNAG, and CAG. **(B)** The Venn map of the OTUs distribution of group OLGIM I and OLGIM II. **(C)** The Venn map of the OTUs distribution of group *H. pylori* positive and *H. pylori* negative.

**Figure 3:**
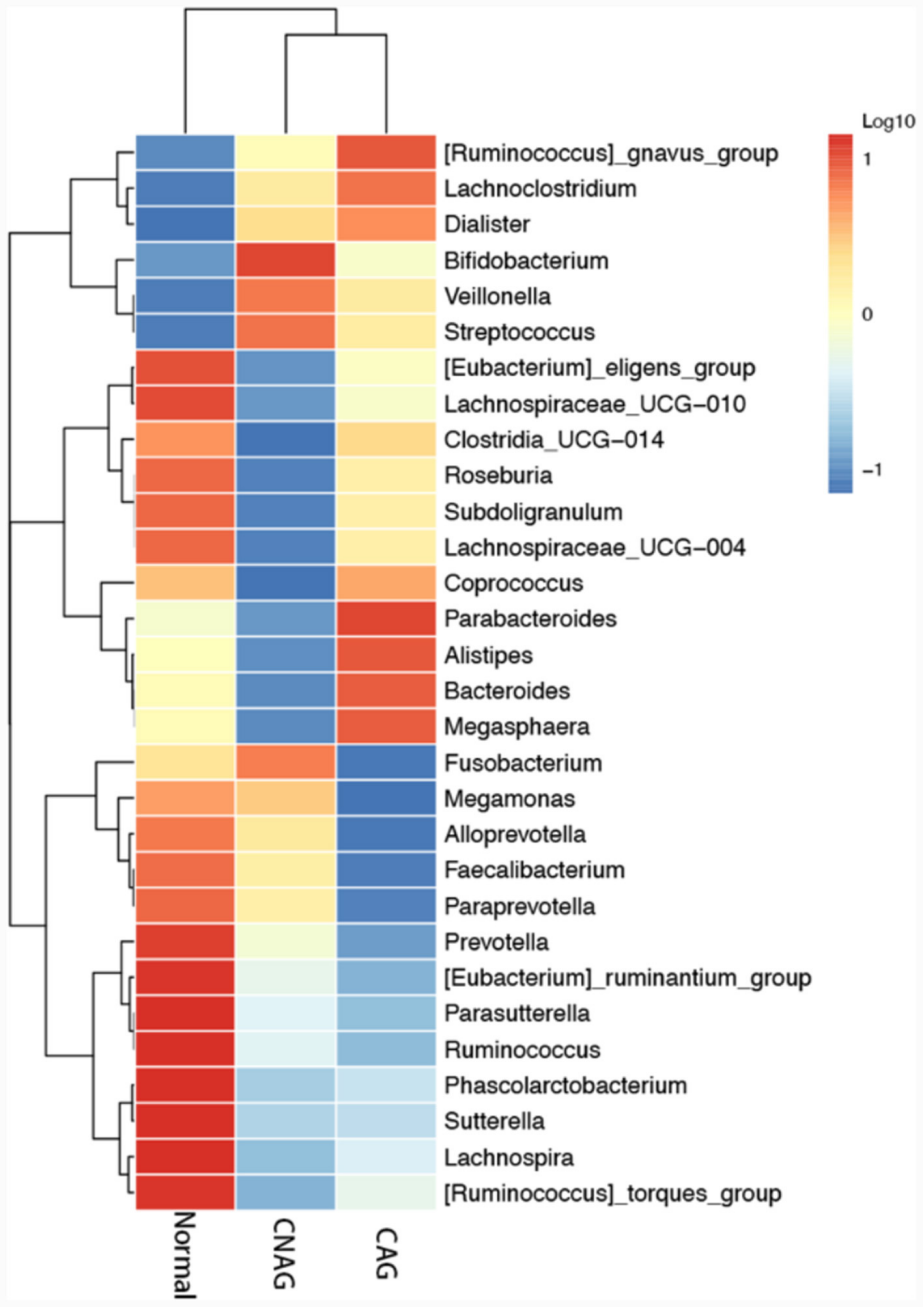
The heatmap of the top 30 genera in group Normal, CNAG, and CAG.

**Figure 4:**
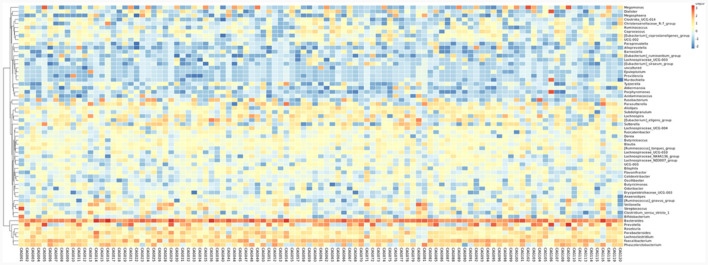
The heatmap of the top 60 genera for all the chronic gastritis subjects.

To profile the gut microbiota linked to the Normal, CNAG, and CAG groups, we conducted 16S rRNA gene sequencing on 176 fecal samples. From the three groups, we identified a total of 5,385, 5,870, and 6,098 OTUs (97% similarity), respectively (Figure 2A). A heatmap was generated to illustrate the top 30 genera within each group (Figure 3). For 105 fecal samples, 5,773 and 5,727 OTUs (97% similarity) were obtained from OLGIM I and OLGIM II group (Figure 2B). The top 60 genera within each group were described in a heat map (Figure 4). Of all the 176 fecal samples, 5,790 and 6,186 OTUs (97% similarity) were obtained from Hp (+) and Hp (–) group (Figure 2C).

**Figure 5:**
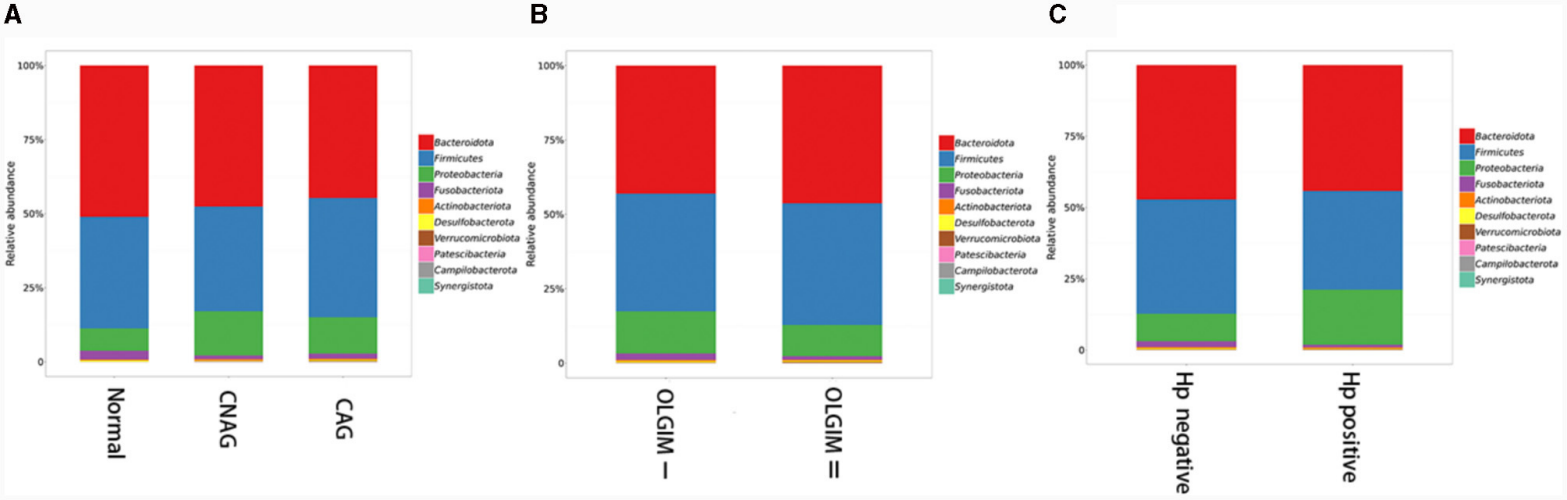
The predominant bacterial phylum distribution composition of each group. **(A)** The predominant bacterial phyla in group Normal, CNAG, and CAG. **(B)** The predominant bacterial phyla in group OLGIM I and OLGIM II. **(C)** The predominant bacterial phyla in group *H. pylori* positive and *H. pylori* negative.

Furthermore, the three predominant bacterial phyla in each group consisted of Bacteroidota, Firmicutes, and Proteobacteria (Figures 5A–C), the top three genera in each group are *Bacteroides, Prevotella*, and *Faecalibacterium* (Supplementary Tables 1–3).

### Characteristics of alpha and beta diversity in gut microbiota

**Figure 6:**
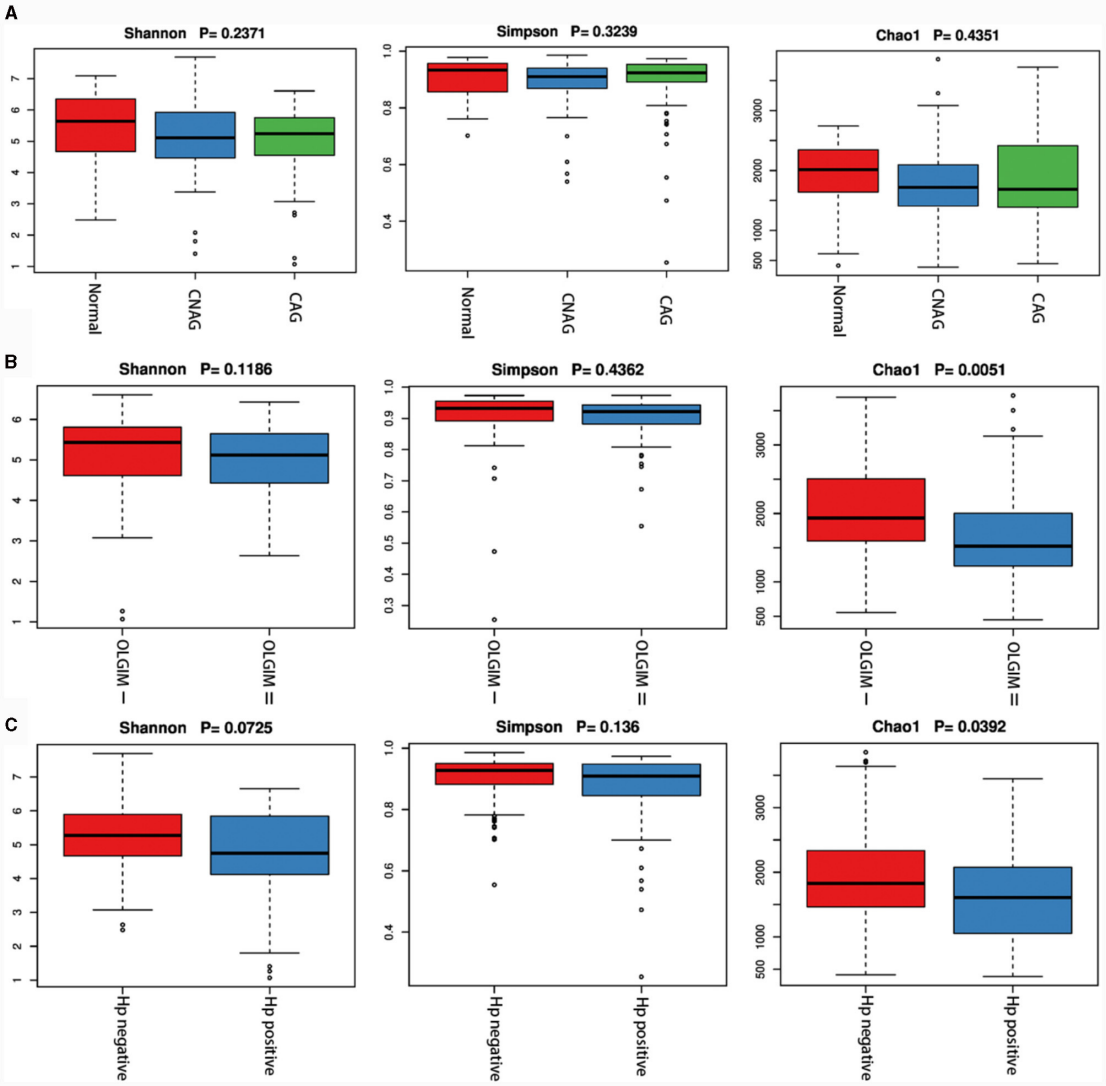
Characteristics of the gut microbiota alpha diversity among each group. **(A)** Characteristics of the gut microbiota alpha diversity among group Normal, CNAG, and CAG. **(B)** Characteristics of the gut microbiota alpha diversity between group OLGIM I and OLGIM II. **(C)** Characteristics of the gut microbiota alpha diversity between group *H. pylori* positive and *H. pylori* negative.

**Figure 7:**
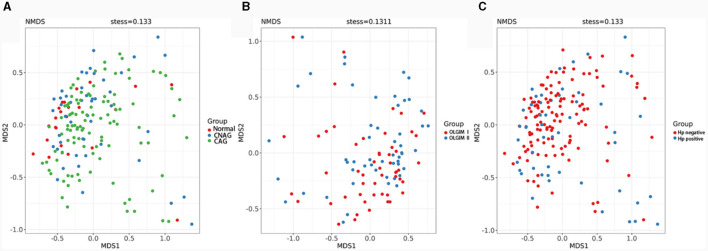
Comparison of the microbiota beta diversity among each group. **(A)** The microbiota beta diversity of group Normal, CNAG, and CAG. **(B)** The microbiota beta diversity of group OLGIM I and OLGIM II. **(C)** The microbiota beta diversity of group *H. pylori* positive and *H. pylori* negative.

We utilized both alpha and beta diversity measures to evaluate gut microbiota dysbiosis across the various groups. Alpha diversity was assessed using community diversity (Simpson indexes and Shannon indexes) and community richness (Chao1 indexes). The Shannon and Simpson indexes of group Normal, CNAG and CAG, group OLGIM I and OLGIM II, group Hp (+) and Hp (–) show no significant differences. Chao1 indexes exhibited a significant decrease in group OLGIM II and group Hp (+) (*P* < 0.05; Figures 6A–C). Non-metric Multidimensional Scaling (NMDS) analyses were carried out to assess beta diversity, allowing us to quantify the dissimilarity in gut microbial composition. The NMDS plots revealed substantial differentiation in the intestinal microbiome across the distinct groups (Figures 7A–C).

### Features of the microbial structure profiles

**Figure 8:**
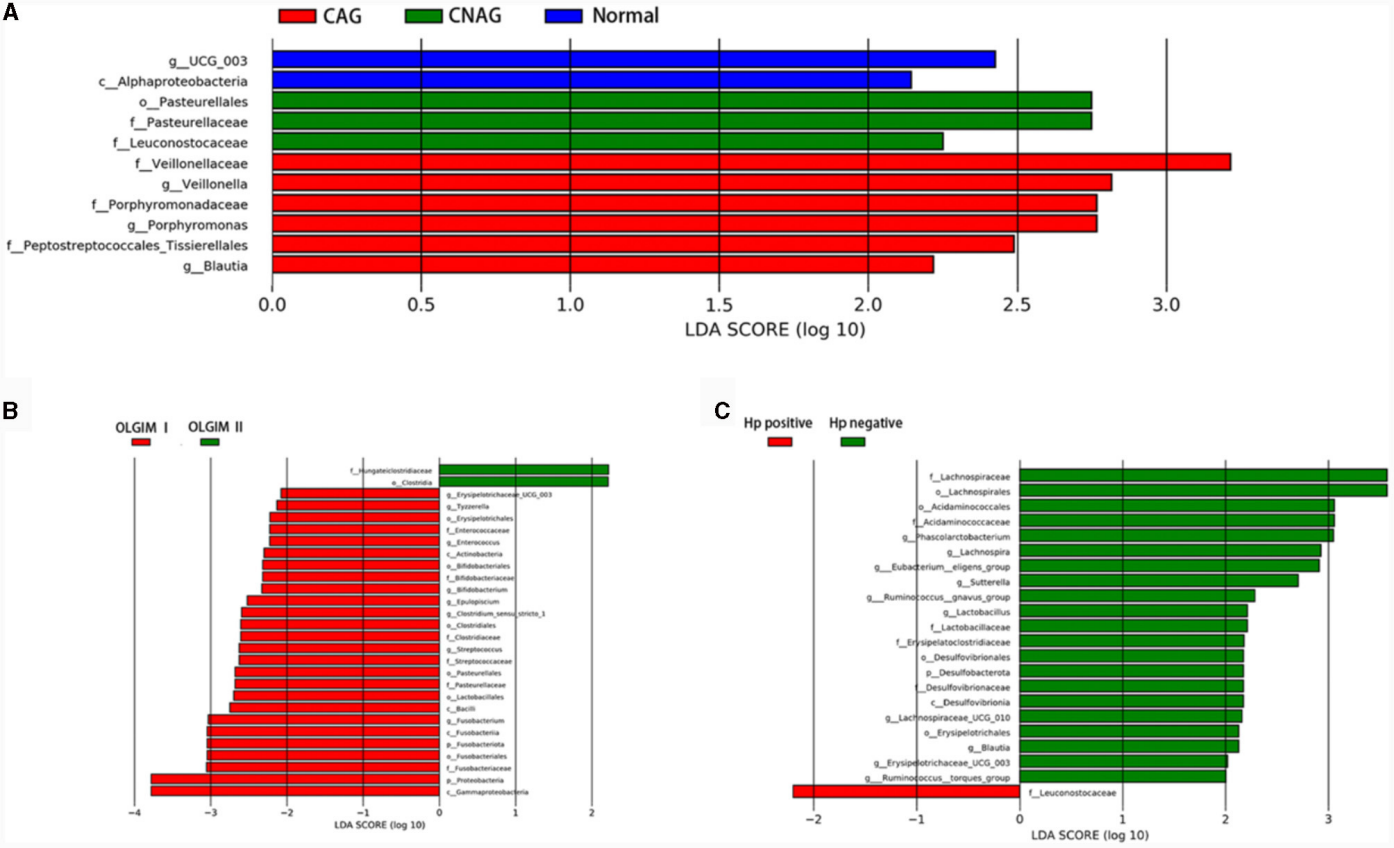
Comparing the distributions of the gut microbiota composition among each group. **(A)** Histogram of LDA value distribution represents the magnitude of the impact of different species among group Normal, CNAG, and CAG. **(B)** Histogram of LDA value distribution represents the magnitude of the impact of different species among group OLGIM I and OLGIM II. **(C)** Histogram of LDA value distribution represents the magnitude of the impact of different species among group *H. pylori* positive and *H. pylori* negative.

To validate the distinctions in microbial communities, we conducted LEfSe analyses, leveraging Linear Discriminant Analysis (LDA) to pinpoint bacteria that displayed significant abundance variations across various groups (LDA score >2.0 with *P* < 0.05). The length of each bar in the chart indicated the extent of influence of various species (LDA Score), signifying the impact of significantly differing species between the various groups (Figures 8A–C).

In total, 11 significant phylotypes were recognized, with 2, 3, and 6 bacterial species predominantly present in the Normal, CNAG, and CAG groups, respectively. At the genus level, *Oscillospiraceae_UCG_003* (*P* = 0.041) exhibited significant enrichment in the Normal group, *Veillonella* (*P* = 0.025), *Porphyromonas* (*P* = 0.044), *Blautia* (*P* = 0.005) exhibited significant enrichment in the CAG group.

In the OLGIM I and OLGIM II groups, there were 26 and 2 species of bacteria that were predominant, respectively. At the genus level, *Erysipelotrichaceae_UCG_003* (*P* = 0.036), *Tyzzerella* (*P* = 0.042), *Enterococcus* (*P* = 0.015), *Bifidobacterium* (*P* = 0.023), *Epulopiscium* (*P* = 0.019), *Clostridium_sensu_stricto_1* (*P* = 0.030), *Streptococcus* (*P* = 0.006), *Fusobacterium* (*P* = 0.030) showed a significant enrichment in the OLGIM I group.

**Figure 9:**
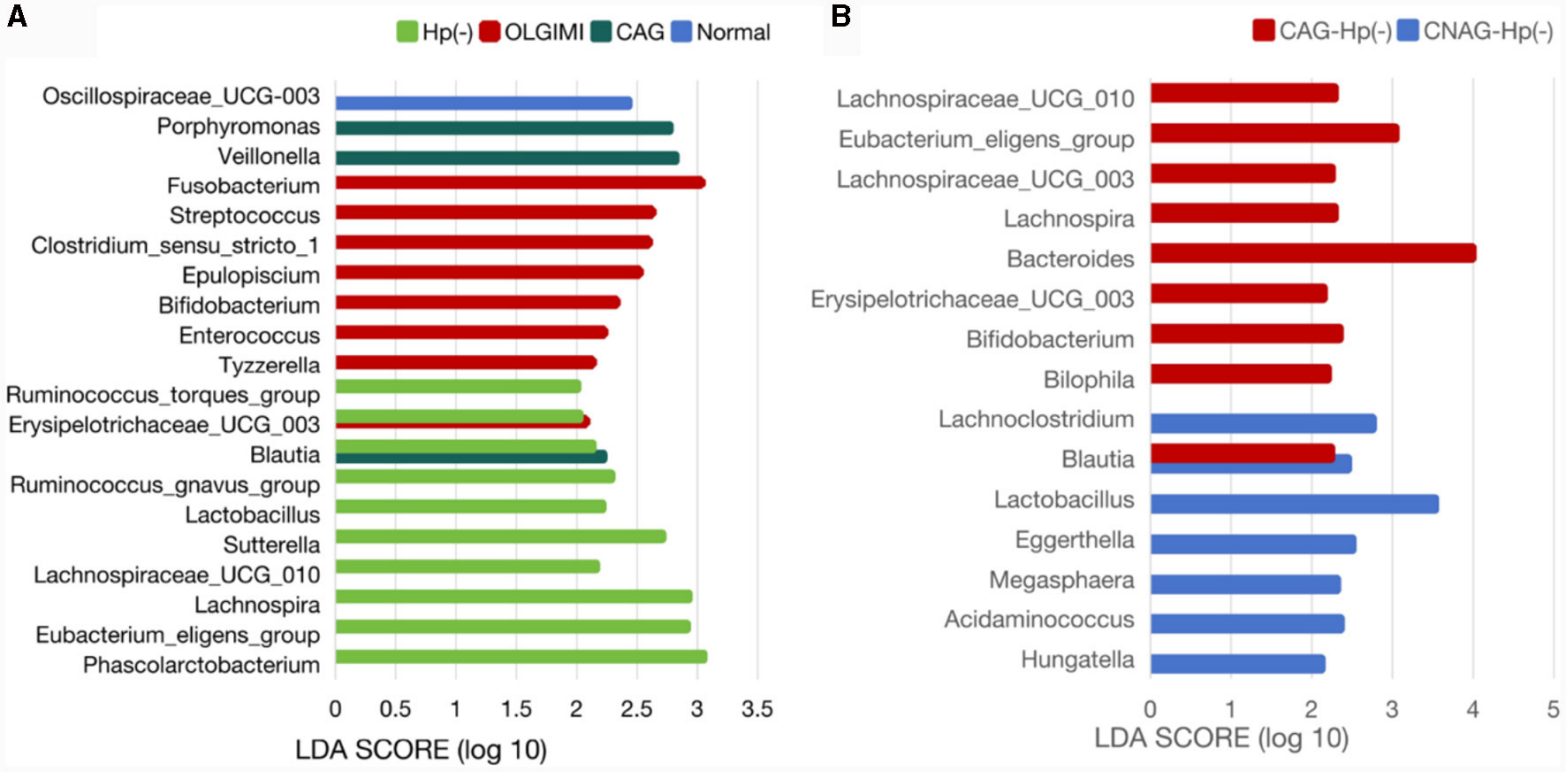
Comparison of the significantly enriched genera in each group. **(A)** The histogram of LDA values represents the impact level of significantly enriched genera in the Normal, CAG, OLGIM I, and Hp (-) group. **(B)** Histogram of LDA value distribution represents the impact level of significantly enriched genera in the CNAG-Hp (-) and CAG-Hp (-) group.

Twenty-two significant phylotypes were identified in Hp (+) and Hp (–) group, 21 and 1, respectively. At the genus level, *Phascolarctobacterium* (*P* = 0.020), *Eubacterium_eligens_group* (*P* = 0.018), *Lachnospira* (*P* = 0.004), *Lachnospiraceae_UCG_010* (*P* = 0.040), *Sutterella* (*P* = 0.030), *Lactobacillus* (*P* = 0.032), *Ruminococcus_gnavus_group* (*P* = 0.049), *Blautia* (*P* = 0.003), *Erysipelotrichaceae_UCG_003* (*P* = 0.024), *Ruminococcus_torques_group* (*P* = 0.042) showed a significant enrichment in the Hp (–) group (Figure 9A).

### Exploration of the relationship between clinical characteristics and gut microbiota

**Figure 10:**
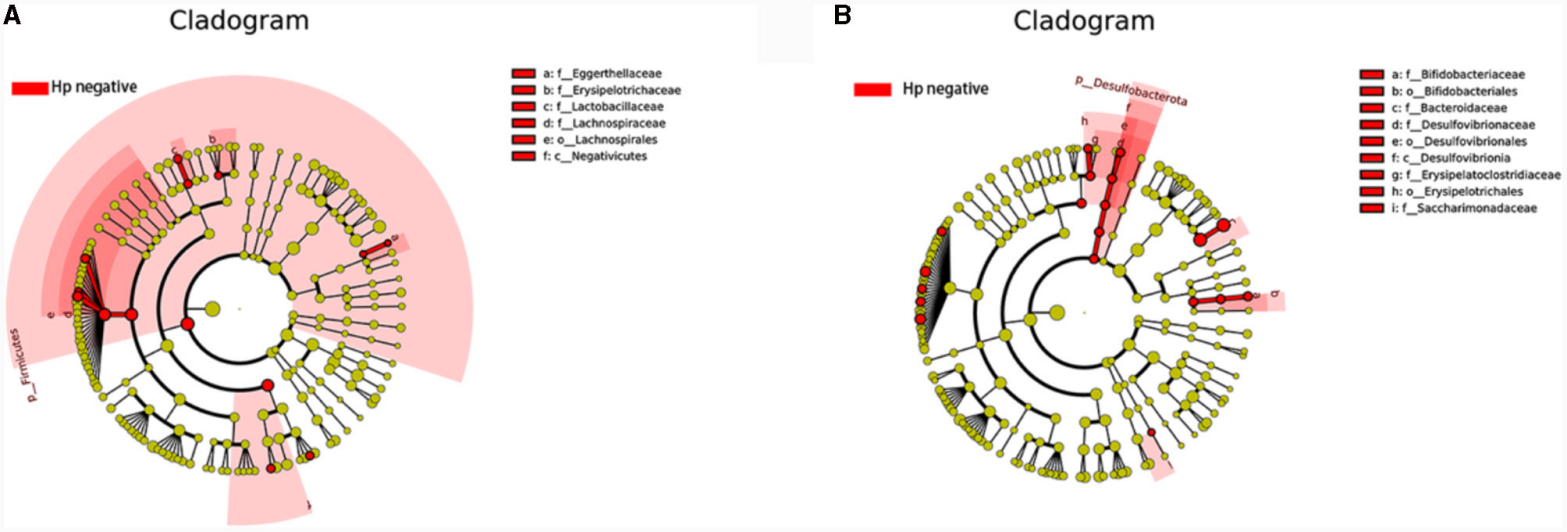
Comparing the distributions of the gut microbiota structure among each group. **(A)** The cladogram illustrates the phylogenetic distribution of microbial lineages between group *H. pylori* positive and *H. pylori* negative among CNAG subjects. **(B)** The cladogram illustrates the phylogenetic distribution of microbial lineages between group *H. pylori* positive and *H. pylori* negative among CAG subjects.

Trough LEfSe analyses, nine species of bacteria were significantly differed between the CNAG and CAG group. In order to explore the connections between gut microbiota and *H. pylori* infection in all subjects with CG, we examined the gut microbiota of individuals with CNAG and CAG, categorizing them into Hp (+) and Hp (–) groups. To be specific, the abundances of *Hungatella* (*P* = 0.0003), *Acidaminococcus* (*P* = 0.0148), *Megasphaera* (*P* = 0.0066), *Eggerthella* (*P* = 0.0288), *Lactobacillus* (*P* = 0.0202), *Blautia* (*P* = 0.0490), and *Lachnoclostridium* (*P* = 0.0304) were notably greater in the Hp (–) group as opposed to the Hp (+) group among CNAG subjects (Figure 10A). The abundances of *Bilophila* (*P* = 0.0089), *Bifidobacterium* (*P* = 0.0371), *Blautia* (*P* = 0.0040), *Erysipelotrichaceae_UCG_003* (*P* = 0.0215), *Bacteroides* (*P* = 0.0304), *Lachnospira* (*P* = 0.0082), *Lachnospiraceae_UCG_003* (*P* = 0.0281), *Eubacterium_eligens_group* (*P* = 0.0077), and *Lachnospiraceae_UCG_010* (*P* = 0.0293) exhibited a marked increase in the Hp (–) group as opposed to the Hp (+) group among CAG subjects (Figures 9B, 10B).

## Discussion

Considering that antibiotic and PPI treatments may exacerbate disturbances in the gut microbiota, we excluded 34 subjects who were undergoing medication treatment and those who refused *H. pylori* examination. In the end, 16S rRNA gene sequencing of feces samples were performed for 176 subjects to determine the characteristics of gut microbiota for Normal, CNAG, and CAG subjects. As well as characteristics of gut microbiota for Hp (+) and Hp (–) group. Through histological stages of CAG, the gut microbiota characteristics of OLGIM I and OLGIM II were also described.

Through basic information comparison of participants in each group, it was found that the age of participants gradually increased from the Normal group to the CNAG group and then to the CAG group. This aligns with the common understanding that the incidence of CAG tends to increase with age (Adamu et al., 2010; Chen Z. et al., 2022). When comparing the occupations between group OLGIM I and group OLGIM II, as well as group Hp (+) and Hp (–) separately, we observed that there was a higher proportion of participants engaged in agriculture in group OLGIM II and group Hp (+). This suggests that engaging in agricultural activities is associated with a higher degree of the OLGIM staging and *H. pylori* infection, which aligns with relevant research results regarding the impact of occupation on the incidence of CAG (Chen Z. et al., 2022). Smoking and alcohol consumption, identified as risk factors for the onset and progression of CG, showed no significant differences in the comparisons among groups in this study, possibly due to the sample size.

The sequencing results of feces samples indicate that, despite no differences in community diversity, there is a significant decrease in community richness in OLGIM II and Hp (+) group. Through NMDS analysis, the stress values for each group were all < 0.2. Which means the results of the NMDS analysis have a certain level of interpretative significance, indicating that beta diversity analyses reveal a noteworthy segregation of gut microbiota among distinct groups.

The genus *Oscillospiraceae_UCG_003* was only significantly enriched in the Normal group, and decreased in other disease like depression (Liang et al., 2022), suggesting that it may be a beneficial bacterial genus for health. Of all the bacterial significantly enriched in Hp (–) group, *Phascolarctobacterium, Eubacterium_eligens_group*, and *Lachnospira* were the top three genera with the highest extent of influence on the group. *Phascolarctobacterium* and *Eubacterium_eligens_group also* showed significant impact on the group Hp (–) group among CAG subjects. *Lactobacillus* was significantly enriched in both the Hp (–) group and CNAG-Hp (–) group, demonstrating a strong extent of influence. Based on the evidence presented, we could assume that these three genera *Eubacterium_eligens_group, Lachnospira*, and *Lactobacillus* might be potentially protective bacterial genera against *H. pylori* infection. *Eubacterium_eligens_group* were also discovered to have a protective effect on other diseases like scoliosis (Lai et al., 2023) and type 1 diabetes (Luo et al., 2023). Lachnospiraceae constitute an integral part of the gut microbiota's core, characterized by their diversity of species and consistent relative abundance throughout the host's lifespan. *Lachnospira* belong to the Lachnospiraceae family and are known for being primary contributors to short-chain fatty acid production. Patients experiencing active ulcerative colitis and exhibiting depression and anxiety tend to have lower richness in genus *Lachnospira* (Yuan X. et al., 2021). Zhang et al. (2022) found that *Lachnospira* were significantly reduced in type 2 diabetes and diabetic nephropathy patients. As a kind of potential probiotics, *Lachnospira* showed a protective effect against *H. pylori* infection in this study. *Lactobacillus* is widely present in the environment, food, and various parts of the human body, constituting a bacterial genus that rarely causes opportunistic infections in the human body (O'Callaghan and O'Toole, 2013). Several species of *Lactobacillus* are commonly employed as probiotics, contributing to the wellbeing of the host when administered in sufficient quantities (Yang et al., 2018). Considering the beneficial effects of these three bacterial genera on health and the demonstrated significant extent of influence on groups which related with negative *H. pylori* infection status to a considerable degree of our study, an increase in bacterial abundance of genera *Eubacterium_eligens_group, Lachnospira*, and *Lactobacillus* might serve as a negative indicator for diagnosing *H. pylori* infection. The abundance of bacterial *Bifidobacterium* was enriched in the OLGIM I group and the CAG-Hp (–) group, which means the gut microbiota characteristics of bacterial *Bifidobacterium* enrichment could be linked to the occurrence of *H. pylori* negative CAG (especially for OLGIM I). *Bifidobacterium* and *Lactobacillus* are two types of probiotics which have been suggested as adjuncts to antibiotics to address *H. pylori* (Boltin, 2016). Though didn't found to be enriched in Normal group, these two genera were corelated with *H. pylori* negative in this study. These evidences also show the negative correlation between *H. pylori* infection and two probiotics *Bifidobacterium* and *Lactobacillus*.

*Veillonella* and *Porphyromonas* exhibited significant enrichment in the CAG group and *Fusobacterium* in OLGIM I group. *Veillonella* species are commonly found in the oral microbiota and have been repeatedly reported to be associated with inflammatory conditions in the oral cavity (Luo et al., 2020; Yuan H. et al., 2021; Giacomini et al., 2023; Kumar et al., 2023). In certain pathological conditions, oral bacteria *Veillonella* can ectopically colonize in the intestine (Rojas-Tapias et al., 2022). Liu et al. (2022) found that the relative abundance of *Veillonella* was richer in the GIM group than in the chronic superficial gastritis group. Hua et al. (2023) observed that *H. pylori* infection reduced the abundances of several predominant oral bacteria in patients with CAG. Additionally, bile reflux markedly facilitated the colonization of dominant oral microbiota, particularly *Veillonella*, in the stomach of CAG patients. The increase in *Veillonella* abundance may serve as one of the indicators for CAG and GIM. Research show that the abundance of *Porphyromonas* in gastritis samples were increased (Chen M. et al., 2022). The abundance of *Porphyromonas* showed a correlation with histological gastritis, but not with endoscopic or symptomatic gastritis (Han et al., 2019). Through testing the variations in *Porphyromonas* abundance may enhance the accuracy of diagnosing gastritis through endoscopy or symptoms. The genus *Fusobacterium* has been found to be closely associated with gastrointestinal diseases. Li's team discovered that, compared to gastritis, the genus *Fusobacterium* is frequently and significantly enriched in GC patients, demonstrating a strong ability to distinguish between GC samples and gastritis (Li et al., 2023). This suggests that the abundance of the genus *Fusobacterium* may be associated with the progression of GIM and CAG. The strong coexcluding interactions between *H. pylori* and *Fusobacterium* might be one reason we didn't observed the richness of *Fusobacterium* in Hp (+) group, which has been proved by Gou's team (Guo et al., 2020).

Bacterial genus *Blautia* was significantly enriched in group CAG and group Hp (–), as well as in group CNAG-Hp (–) and group CAG-Hp (–). *Blautia* represents a genus of anaerobic bacteria recognized for its probiotic attributes (Liu et al., 2021). Recent researches have demonstrated that variations in the composition and levels of *Blautia* in the gut play a beneficial role in countering colorectal cancer (Ni et al., 2022), depressive disorder (Zhuang et al., 2020), type 2 diabetes and obesity (Hosomi et al., 2022). Zhou et al. (2020) observed that the bacterial *Blautia* decreased in gastritis subjects. Huang et al.'s (2022) team found that the abundance of bacteria *Blautia* decreased in CAG patients. These studies have shown the beneficial effects of the bacteria on health. Indeed, the presence of *Blautia* in the gut microbiome is influenced by factors such as host age, geographic location, dietary habits, genotype, health, disease status, and various physiological conditions. Notably, this genus comprises 20 newly discovered species with officially recognized names (Nakayama et al., 2015; Odamaki et al., 2016; Mao et al., 2018). In our research, *Blautia* exhibited notable enrichment in four groups. The dominance of a specific *Blautia* species remains uncertain, indicating that it may not be suitable as a diagnostic marker of CAG.

Helicobacter pylori infection, CG, and the composition of the gut microbiota mutually influences each other. The changes in the abundance of gut bacterial genera could reflect *H. pylori* infection and the progression of CG of subjects. For these reasons, learn more about the relationship of significant characteristics of gut microbiota for healthy individuals, CNAG, and CAG (regardless of the etiology, the diagnosis of atrophic gastritis OLGIM I and OLGIM II was confirmed by histopathology) subjects, as well as infection of *H. pylori* at different stage of the disease, might let us learn more about the disease and further identify diagnosis and treatment. Through our research, we have observed that several gut bacterial genera could potentially serve as diagnostic markers for *H. pylori* infection and the progression of CG. This may offer a pathway for gut microbiota detection to reduce the potential incidence of CAG and *H. pylori* infection. However, our study was conducted in a specific region with limited samples, reflecting characteristics of populations in areas with a high prevalence of CAG and *H. pylori* infection, such as Jinjiang City in Fujian Province, China, and similar coastal regions. Further research is needed to uncover universal characteristics in populations.

## Conclusion

We found the gut microbiota characteristics of healthy, CNAG, and CAG subjects have a significant difference, and age of subjects increased from Normal, CNAG to CAG group. There were more farmers in OLGIM II and Hp (+) group. Also, the gut microbiota characteristics of group OLGIM I and OLGIM II, group Hp (+) and Hp (–) show significant differences. Through the analysis of *H. pylori* infection in CNAG and CAG groups, we found the gut microbiota characteristics of different group show significant difference because of *H. pylori* infection. Several bacterial genera showed a significant extent of influence on corresponding group and could be potential diagnostic markers for *H. pylori* infection and the progression of CG. These discoveries may enhance our comprehension of CG, *H. pylori* infection, and gut microbiota. However, to obtain more universally applicable characteristics of the gut microbiota in populations related to gastritis, further research is necessary.

## Data availability statement

The datasets presented in this study are deposited in the NCBI repository under accession number PRJNA1072256 (https://www.ncbi.nlm.nih.gov/sra/PRJNA1072256).

## Ethics statement

The studies involving humans were approved by Jinjiang Hospital of Traditional Chinese Medicine, affiliated with Fujian University of Traditional Chinese Medicine Medical Ethics Committee. The studies were conducted in accordance with the local legislation and institutional requirements. The participants provided their written informed consent to participate in this study.

## Author contributions

HL: Conceptualization, Data curation, Formal Analysis, Investigation, Methodology, Visualization, Writing – original draft. YHu: Formal Analysis, Investigation, Methodology, Validation, Visualization, Writing – original draft. YHua: Investigation, Resources, Software, Supervision, Visualization, Writing – review & editing. SD: Resources, Software, Supervision, Validation, Writing – review & editing. LZ: Formal Analysis, Investigation, Supervision, Validation, Writing – review & editing. XLi: Project administration, Resources, Validation, Writing – review & editing. ML: Resources, Software, Validation, Writing – review & editing. WH: Data curation, Resources, Validation, Writing – review & editing. XLin: Conceptualization, Funding acquisition, Investigation, Methodology, Supervision, Validation, Visualization, Writing – review & editing.
